# Adaptogenic potential of royal jelly in liver of rats exposed to chronic stress

**DOI:** 10.1371/journal.pone.0191889

**Published:** 2018-01-29

**Authors:** Douglas Carvalho Caixeta, Renata Roland Teixeira, Leonardo Gomes Peixoto, Helen Lara Machado, Nathalia Belele Baptista, Adriele Vieira de Souza, Danielle Diniz Vilela, Celso Rodrigues Franci, Foued Salmen Espindola

**Affiliations:** 1 Institute of Biotechnology, Federal University of Uberlândia, Uberlândia, Minas Gerais, Brazil; 2 Department of Physiology, University of São Paulo, Ribeirão Preto, Minas Gerais, Brazil; Stellenbosch University, SOUTH AFRICA

## Abstract

Restraint and cold stress increase both corticosterone and glycemia, which lead to oxidative damages in hepatic tissue. This study assessed the effect of royal jelly (RJ) supplementation on the corticosterone level, glycemia, plasma enzymes and hepatic antioxidant system in restraint and cold stressed rats. Wistar rats were allocated into no-stress, stress, no-stress supplemented with RJ and stress supplemented with RJ groups. Initially, RJ (200mg/Kg) was administered for fourteen days and stressed groups were submitted to chronic stress from the seventh day. The results showed that RJ supplementation decreases corticosterone levels and improves glycemia control after stress induction. RJ supplementation also decreased the body weight, AST, ALP and GGT. Moreover, RJ improved total antioxidant capacity, SOD activity and reduced GSH, GR and lipoperoxidation in the liver. Thus, RJ supplementation reestablished the corticosterone levels and the hepatic antioxidant system in stressed rats, indicating an adaptogenic and hepatoprotective potential of RJ.

## Introduction

The adaptive response to stress is characterized by psychophysiological adaptations of an organism to restore homeostasis [1]. It is well documented that chronic restraint and cold stress effectively mimics physical and psychological stress [2], elevate metabolic rate and also increase production of reactive oxygen species (ROS). Physical and psychological stress activates the hypothalamic-pituitary-adrenal (HPA) axis and the autonomic nervous system (ANS), increasing plasma glucocorticoid levels [3]. Increased corticosterone levels were observed in stress responses using the stress models, such as restraint and cold [2], immobilization [4], cold [5], cold water immersion [5], electric foot shock [6] and social isolation stress [7]. Corticosterone increases gluconeogenesis and hepatic glycogenolysis in rats, resulting in an increase in the availability of metabolic substrates [8].

A metabolic byproduct of stress-induced increase in energy production is the formation of ROS [9] e.g. hydrogen peroxide (H_2_O_2_), hydroxyl radicals (HO^·^) and superoxide anion radicals (O2^–·^), which cause lipid peroxidation. In addition, the increased corticosterone levels trigger ROS production and promote redox imbalance in different tissues of the body [10, 11]. Oxidative stress also associated with physical stress induces changes in antioxidant defense systems [11]. Therefore, to neutralize reactive oxygen species, the body uses mainly enzymatic and non-enzymatic antioxidant defense system [12].

Several studies have investigated whether nutraceutical supplementation results in enhancement of the antioxidant defense system. In relation to stress management, nutraceutical supplements have been used as an adaptogenic agent to support the body’s adaptation in stressful situations [13]. Thus, royal jelly (RJ) could be used as a nutraceutical product. RJ is secreted by the mandibular and hypopharyngeal glands of worker bees (*Apis mellifera* L.). Royal Jelly composition includes major royal jelly proteins, free amino acids, sugars, vitamins (B1, B2, B6, folic acid, pantothenic acid, nicotinic acid, and biotin) and lipids such as 10-hydroxy-2-decenoic acid (HDA-10). The RJ has several biological properties including anti-inflammatory [14], vasodilator and hypotensive [15], antimicrobial [16], immunomodulatory [17], hypocholesterolemic [18] and antioxidant [19] activities.

In a previous study from our research group, we demonstrated a neuroprotective effect of royal jelly supplementation and a reduction of corticosterone levels in a stress condition [20]. With these interesting results, the interest arose to evaluate the effect of the royal jelly supplementation on the liver, which is a central organ of metabolism and is related to the synthesis of cholesterol, a precursor of corticosterone. As well as, to investigate the effect of RJ supplementation on a non-stressful situation. As glucocorticoids have direct and indirect modulatory roles in oxidative stress [9], our hypothesis was that RJ could decrease corticosterone levels, even in the absence of stress, and oxidative stress in liver tissue. Thus, the aim of the study was to evaluate the adaptogenic and antioxidant effect of RJ supplementation in rats submitted to chronic stress induced by restraint and cold.

## Materials and methods

### Samples

RJ was imported from China and provided by Apiário Girassol Ltda. (Uberlândia—MG, Brazil). The centesimal analysis of the royal jelly was carried out by the Laboratory of Bromatology and Animal Nutrition of the Federal University of Uberlândia. The royal jelly has 67% of humidity and 33% of dry matter, of these, 14.19% are crude protein, 2.01% are lipids, 0.87% are ash and 15.93% are carbohydrates. The RJ was stored at -20°C until use. Daily, 200 mg/kg b.w. of RJ samples were prepared for use in supplementation.

### Animals

Wistar rats (207–250g) were obtained and kept in the Center for Bioterism and Experimentation at the Federal University of Uberlândia, Uberlândia, Brazil. Animals were kept in controlled conditions (22 ± 1 °C, humidity 60% ± 5 and 12-hour light-dark cycles– 6:00/18:00 h lights on/off) with a standard diet and water *ad libitum*. Body weight was measured at the beginning and end of the study, while water and food intake per animal were measured daily. All experimental procedures were approved and conducted by the Brazilian Society of Laboratory Animal Science and the Ethics Committee for Animal Research of the Federal University of Uberlândia, Brazil (CEUA No. 047/14).

### Induction of stress by restraint and cold

Animals were randomly allocated into four groups (n = 10/group): no stress (NS); no stress supplemented with royal jelly (NSRJ); stress (S); and stress supplemented with royal jelly (SRJ). The animals were exposed to stress by restraint and cold, using the method of Paula-Freire et al. [21]. These method effectively mimics a condition of physical and psychological stress [2]. The restraint stress was carried out using individual acrylic hemicylindrical plastic tubes (4.5 cm diameter, 12 cm long) for 2 hours daily in the morning (8:00 h– 10:00 h.). The cold stress was carried at 10°C for 2 hours daily in the afternoon (16:00 h– 18:00 h).

Supplementation with RJ began seven days before the stress sessions with subsequent supplementation for seven more days during the period of stress induction [21]. RJ was administered by oral gavage 45 minutes before the stress session. The NS and S rats received a placebo (water) and the NS and NSRJ rats were not exposed to stressors.

On the fourteenth day, animals were exposed to the two stressors simultaneously for 2 hours [21] until euthanasia. Glucose levels were measured before and after this last session of stress (Sb—stress before; SRJb—stress supplemented with RJ before stress session; Sa—stress after; SRJa—stress supplemented with RJ after stress session) by puncturing the tail vein, using reactive strips (Accu-Chek Performa, Roche Diagnostic Systems, Basel, Switzerland).

The NS, NSRJ, S, SRJ rats were anaesthetized with ketamine (90 mg/kg) and xylazine (20 mg/kg), in accordance the methods of Arnold and Langhans, 2010 [22]. All groups were subjected to the same manipulation procedure. Plasma samples were used to assess corticosterone levels and hepatic enzyme activities, whereas liver tissues were used for oxidative stress analysis.

### Determination of corticosterone levels by radioimmunoassay

Blood samples were collected (08:00 h– 11:00 h in heparinized plastic tubes and centrifuged at 1200g at 4°C for 15 min.) after the last session of stress via cardiac puncture in the right ventricle. Plasma was separated and frozen at -20°C until the assay. Radioimmunoassay (RIA) used H3-corticosterone from NEN Life Science Products (Boston, USA) and a standard reference specific antibody from Sigma (St. Louis, MO, USA). Corticosterone was used to measure tritiated recovery [23]. The intra-assay error was 4.5% and the minimum detectable dose was 0.08 ng/ml.

### Hepatic enzymes activities in plasma

Aspartate transaminase (AST), alanine transaminase (ALT), γ-glutamyl transferase (GGT) and alkaline phosphatase (ALP) were measured at the Laboratory of Clinical Analyses, School of Veterinary Medicine, Federal University of Uberlândia, using an automatic analyzer (Cobas Mira, Roche Diagnostic Systems, Basel—Switzerland), by using commercial kits (Labtest Diagnóstica, Lagoa Santa—Brazil).

### Sample collection and tissue preparation

The liver tissues were quickly removed, separated in lobes, washed (NaCl 0.9% buffer) and immersed in liquid nitrogen. Then, the same part of liver tissues were thawed and homogenized in phosphate buffer (1:10 w/v, pH 7.4). The homogenates were centrifuged at 800 x g for 15 min at 4°C, and the total protein concentration in the supernatant samples was measured, according to the Bradford assay [24].

### Oxidative stress marker analysis

#### Thiobarbituric acid reactive substances (TBARS)

Lipid peroxidation was measured by the reaction between malondialdehyde in the liver samples (MDA) and thiobarbituric acid (0.67% TBA). Organic-phase fluorescence was evaluated at 515 nm (excitation) and at 553 nm (emission). A MDA standard curve allowed the quantification of the compound in the samples by linear regression [25]. TBARS levels were calculated as nmol TBARS/mg of protein.

#### Total antioxidant capacity (FRAP)

Total antioxidant capacity was evaluated by the capacity of the samples to reduce Fe^+3^ to Fe^+2^, which was then chelated by TPTZ (2,4,6-Tris(2-pyridyl)-s-triazine) in order to form the deep-blue colored Fe^+2^-TPTZ complex [25]. This complex was measured in a spectrophotometer at 593 nm.

#### Superoxide dismutase (SOD) activity

SOD activity was measured by the inhibition autoxidative capacity of pyrogallol. The SOD activity was evaluated using a spectrophotometer at 420 nm. A calibration curve was constructed using SOD as standard. A 50% inhibition of autoxidation of pyrogallol was defined as one SOD unit [25].

#### Reduced glutathione (GSH)

The protein content of the samples was initially precipitated by metaphosphoric acid (MPA) at the ratio of 1:1 (homogenate/MPA). The samples were centrifuged at 7000xg for 10 minutes. The supernatant was collected and mixed with sodium phosphate buffer (100 mM, pH 8.0), containing EDTA (5mM) and ortho-phthaldialdehyde (1 mg/mL in methanol). The mixture was incubated in the dark at room temperature for 15 min and fluorescence was measured at 350 nm (excitation) and 420 nm (emission). A standard curve of GSH (0.001–0.1 mM) was used for linear regression [25].

#### Glutathione peroxidase (GPx) activity

To measure the glutathione peroxidase activity, the homogenate was incubated with GPx buffer (100 mM potassium phosphate containing 1 mM EDTA, pH7.7), sodium azide (40 mM), GSH (diluted in 5% metaphosphoric acid), GR (diluted in GPx buffer), NADPH (diluted with sodium bicarbonate 5%) and tert-butyl (0.5 mM). The reduction in NADPH concentration was evaluated for 10 minutes in a spectrophotometer, at 340 nm [25].

#### Glutathione reductase (GR) activity

GR activity was evaluated using oxidized glutathione (GSSG) and nicotinamide adenine dinucleotide phosphate (NADPH) as substrates. The activity of the enzyme was determined using sodium phosphate buffer (200 mM, pH 7.5), EDTA (6.3 mM), GSSG (1 mM), NADPH (1 mM) and the samples [25]. The consumption of NADPH was measured at 340 nm for 10 minutes. A GR unit is defined as one μmol of reduced GSSG per minute. The specific activity was calculated as U/mg of protein.

#### Glucose-6-phosphate dehydrogenase (G6PDH) activity

The activity of glucose-6-phosphate dehydrogenase was monitored by the production of NADPH with a consequent increase in absorbance at 340nm. The samples were incubated with Tris-HCl buffer (100mM, pH 7.5), magnesium chloride (MgCl_2_, 2 M), NADP^+^ (0.5 mM) and glucose-6-phosphate (1mM). The kinetic readings were monitored for ten minutes [25].

### Statistical analyses

Data were used as independent variables (stress by restraint and cold) and as the dependent variables (body weight, water and food intake, biochemical parameters and oxidative stress). The data were analyzed using the one-way analysis of variance (ANOVA) followed by the Tukey Multiple Comparison as a *post-hoc* test. All analyses were performed using the software GraphPad Prism (GraphPad Prism version 6.00 for Windows; GraphPad Software, San Diego, CA, USA). Outliers were detected by performing Grubb’s test using an online GraphPad outlier calculator (http://graphpad.com/quickcalcs/Grubbs1.cfm). Only values of p < 0.05 were considered significant. Results were expressed as mean ± SEM.

## Results

Table 1 shows the effect of RJ supplementation on body weight, water and food intake and hepatic enzyme activities in the plasma of restraint and cold stressed rats. S, NSRJ and SRJ decreased the body weight compared to NS (F_3, 33_ = 7.757, p < 0.05, F_3, 33_ = 7.757, p < 0.05; F_3, 33_ = 7.757, p < 0.001, respectively), and no change was observed between S and SRJ rats. No significant difference was observed in the water or food intake among the groups. In addition, NSRJ and SRJ decreased the AST levels compared to NS (F_3, 29_ = 17.64, p < 0.001; F_3, 29_ = 17.64, p < 0.01, respectively) and SRJ compared to S rats (F_3, 29_ = 17.64, p < 0.05), while ALT was not different among the groups. S rats had increased GGT compared to NS (F_3, 27_ = 14.17, p < 0.001) whereas SRJ rats had decreased GGT levels compared to S rats (F_3, 27_ = 14.17, p < 0.05). Furthermore, NSRJ and SRJ had decreased ALP levels compared to NS rats (F_3, 31_ = 5.203, p < 0.01; F_3, 31_ = 5.203, p < 0.05, respectively), whereas no change was observed in SRJ compared to S rats or in S compared to NS rats.

**Table 1:** The effect of the stress-induction by restraint and cold and supplementation of royal jelly on body weight, water intake, food intake and hepatic enzymes activities in plasma.

Parameters	NS	NSRJ	S	SRJ
**Δ Body weight (g)**	51.11±2.54	38.97±2.04*	39.63±4.19*	32.22±2.14*
**Water intake (ml)**	41.25±4.19	44.07±4.66	37.56±2.52	38.91±6.25
**Food intake (g)**	23.51±0.35	24.30±0.71	22.98±0.40	22.82±0.27
**AST (U/L)**	99.78±3.30	67.13±5.41*	97±1.19	83.44±3.24*#
**ALT (U/L)**	47.5±4.03	46.38±2.19	48.22±1.66	53.44±2.91
**GGT (U/L)**	8.71±1.42	7.02±0.70	22.49±3.25*	14.16±1.59#
**ALP (U/L)**	336.2±38.04	223±16.35*	279±8.60	242.3±16.89*

Note: Values are expressed as mean ± S.E.M (n = 10).

*p < 0. 05 vs NS rats;

^#^p < 0. 05 vs S rats.

No stress (NS), No stress supplemented with royal jelly (NSRJ), Stress(S) and Stress supplemented with royal jelly (SRJ); Aspartate transaminase (AST); Alanine transaminase (ALT); γ-glutamyl transferase (GGT) and Alkaline phosphatase (ALP).

**Fig 1:**
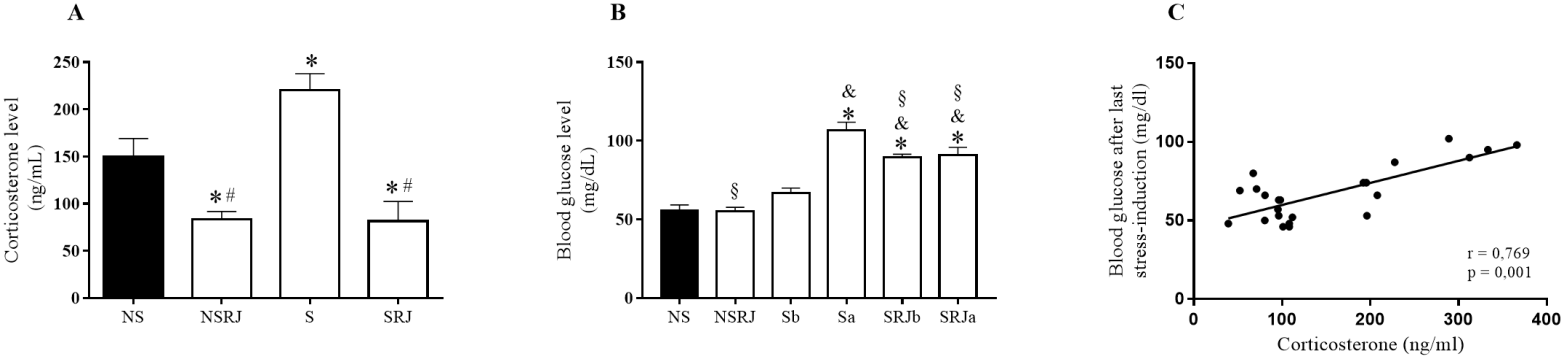
Biomarkers of chronic stress in liver tissue of rats stressed by restraint and cold. Plasma corticosterone level after seven days of stress-induction (A). No stress (NS), No Stress supplemented with Royal Jelly (NSRJ), Stress (S) and Stress supplemented with Royal Jelly (SRJ). Blood glucose level before and after the last stress induction (B). No stress (NS), No Stress supplemented with Royal Jelly (NSRJ), Stress group before the last stress session (Sb), Stress group after the last stress session (Sa), Stress group supplemented with RJ before the last stress session (SRJb), Stress group supplemented with RJ after the last stress session (SRJa). Pearson correlation of mean values of corticosterone levels and blood glucose after the last stress-induction (C). Values are expressed as means ± SEM. *p < 0.05 vs NS, # p < 0.05 vs S, & p < 0.05 vs Sb, § p < 0.05 vs Sa (One-way ANOVA followed by Tukey test). Outliers were detected by performing Grubb’s test using an online GraphPad outlier calculator (http://graphpad.com/quickcalcs/Grubbs1.cfm).

Stress biomarkers were evaluated in plasma samples of NS, NSRJ, S and SRJ rats. Plasma corticosterone levels increased in S compared to NS rats (F_3,28_ = 14.56, p < 0.05) whereas RJ supplementation (NSRJ and SRJ) decreased corticosterone levels compared to NS (F_3,28_ = 14.56, p < 0.05) and S rats (F_3,28_ = 14.56, p < 0.001) (Fig 1A). Furthermore, blood glucose levels did not differ in NSRJ and Sb compared to NS rats, whereas an increase was verified in Sa, SRJb and SRJa compared with both NS (F_5, 47_ = 32.55, p < 0.001) and between Sa and SRJa compared to Sb rats (F_5, 47_ = 32.55, p < 0.001; F_5, 47_ = 32.55, p < 0.01, respectively). When glycemia was compared among the stressed rats, SRJa (F_5, 47_ = 32.55, p < 0.05) and SRJb (F_5, 47_ = 32.55, p < 0.01) had decreased blood glucose levels compared with Sa rats, whereas no difference was observed between SRJb compared to Sb and SRJa rats (Fig 1B). In addition, Pearson correlation between corticosterone levels and glycemia after the last stress induction showed a strong positive correlation (r = 0.769, p = 0.001) (Fig 1C).

**Fig 2:**
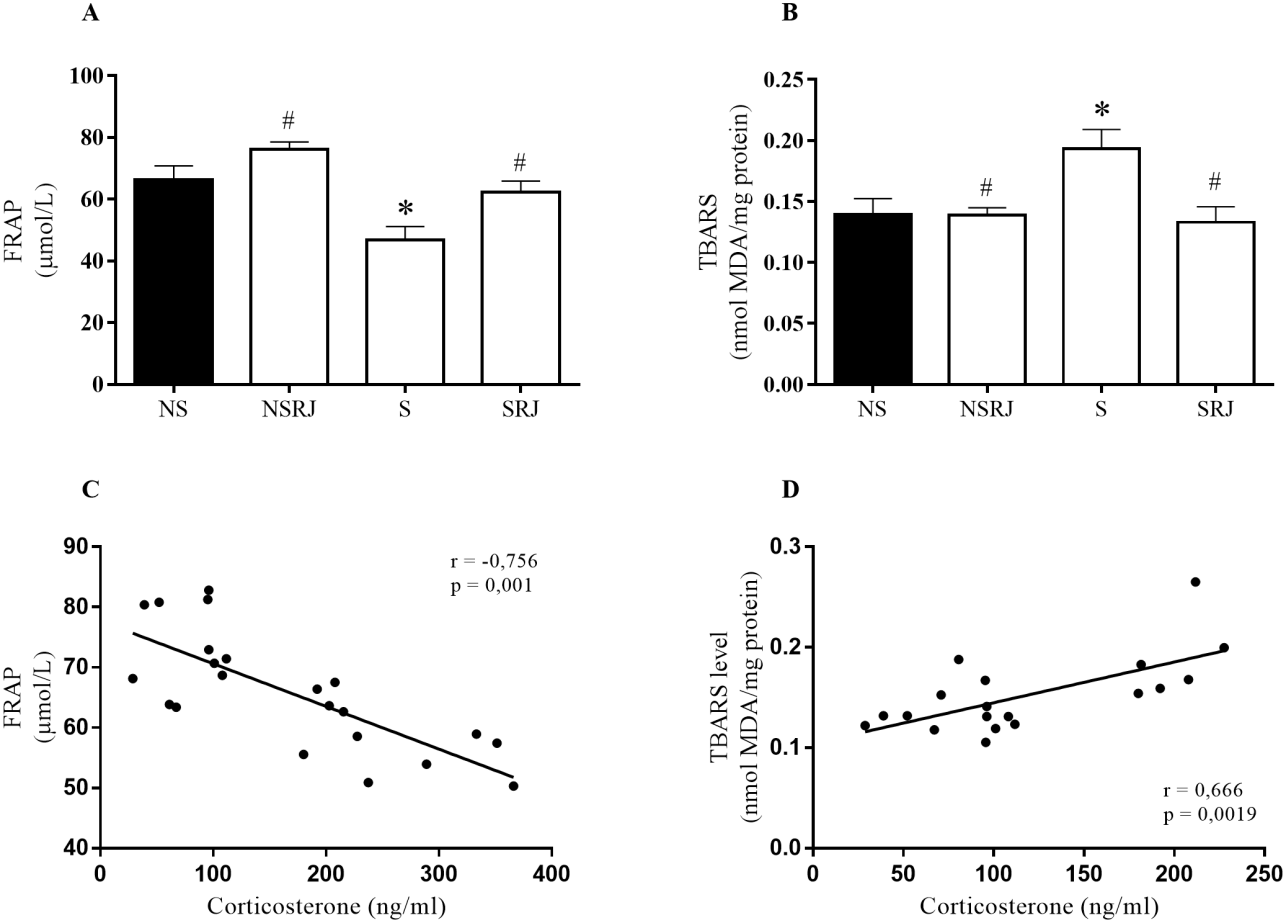
Biomarkers of oxidative stress in liver tissue of rats stressed by restraint and cold. Total antioxidant capacity by FRAP method (A). Lipid Peroxidation by TBARS method (B). No stress (NS), No Stress supplemented with Royal Jelly (NSRJ), Stress (S) and Stress supplemented with Royal Jelly (SRJ). Values are expressed as mean±SEM, * p < 0.05 vs. NS, # p < 0.05 vs. S (One-way ANOVA followed by Tukey test). Pearson correlation of FRAP and TBARS (panels C and D) and means values of corticosterone levels. Outliers were detected by performing Grubb’s test using an online GraphPad outlier calculator (http://graphpad.com/quickcalcs/Grubbs1.cfm).

Fig 2 shows the oxidative stress status in liver tissue. Stressed rats (S) decreased the total antioxidative capacity (FRAP) (F_3, 23_ = 11.65, p < 0.01) and increased lipid peroxidation (TBARS) (F_3, 21_ = 5.027, p < 0.05) compared to NS rats. SRJ rats increased FRAP (F_3, 23_ = 11.65, p < 0.05) and decreased malondialdehyde levels (F_3, 21_ = 5.027, p < 0.05) compared to S rats (Fig 2A and 2B). Pearson correlation between corticosterone and FRAP showed a negative correlation (r = -0.756, p = 0.001), whereas a positive correlation (r = 0.666, p = 0.0019) was observed between corticosterone and lipid peroxidation (Fig 2C and 2D).

**Fig 3:**
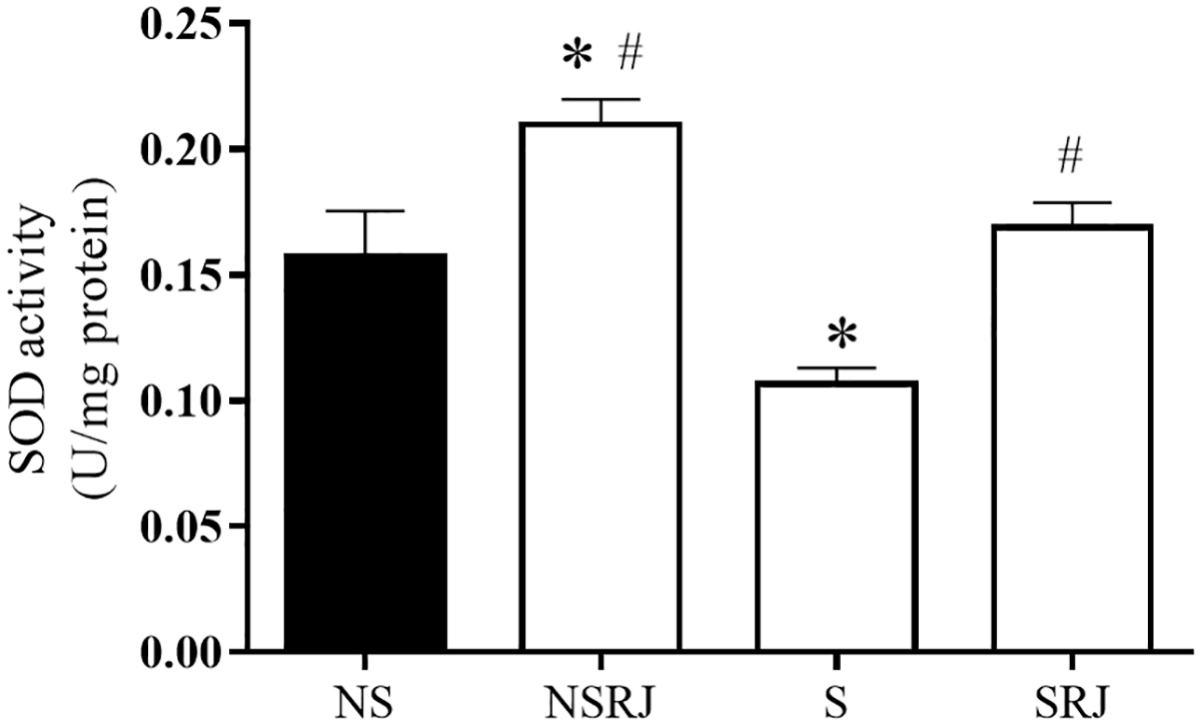
Superoxide dismutase activity in liver tissue of rats stressed by immobilization and cold. No stress (NS), No Stress supplemented with Royal Jelly (NSRJ), Stress (S) and Stress supplemented with Royal Jelly (SRJ). Values are expressed as mean±SEM, * p < 0.05 vs. NS, # p < 0.05 vs. S (One-way ANOVA followed by Tukey test). Outliers were detected by performing Grubb’s test using an online GraphPad outlier calculator (http://graphpad.com/quickcalcs/Grubbs1.cfm).

SOD enzyme activity in liver tissues is shown in Fig 3. The S rats displayed a decreased SOD activity compared to NS rats (F_3, 40_ = 18.18, p < 0.05), whereas RJ supplementation increased the SOD activity in SRJ groups compared to S rats (F_3, 40_ = 18.18, p < 0.001). Furthermore, NSRJ rats increased the SOD compared to NS (F_3, 40_ = 18.18, p < 0.05) (Fig 3).

**Fig 4:**
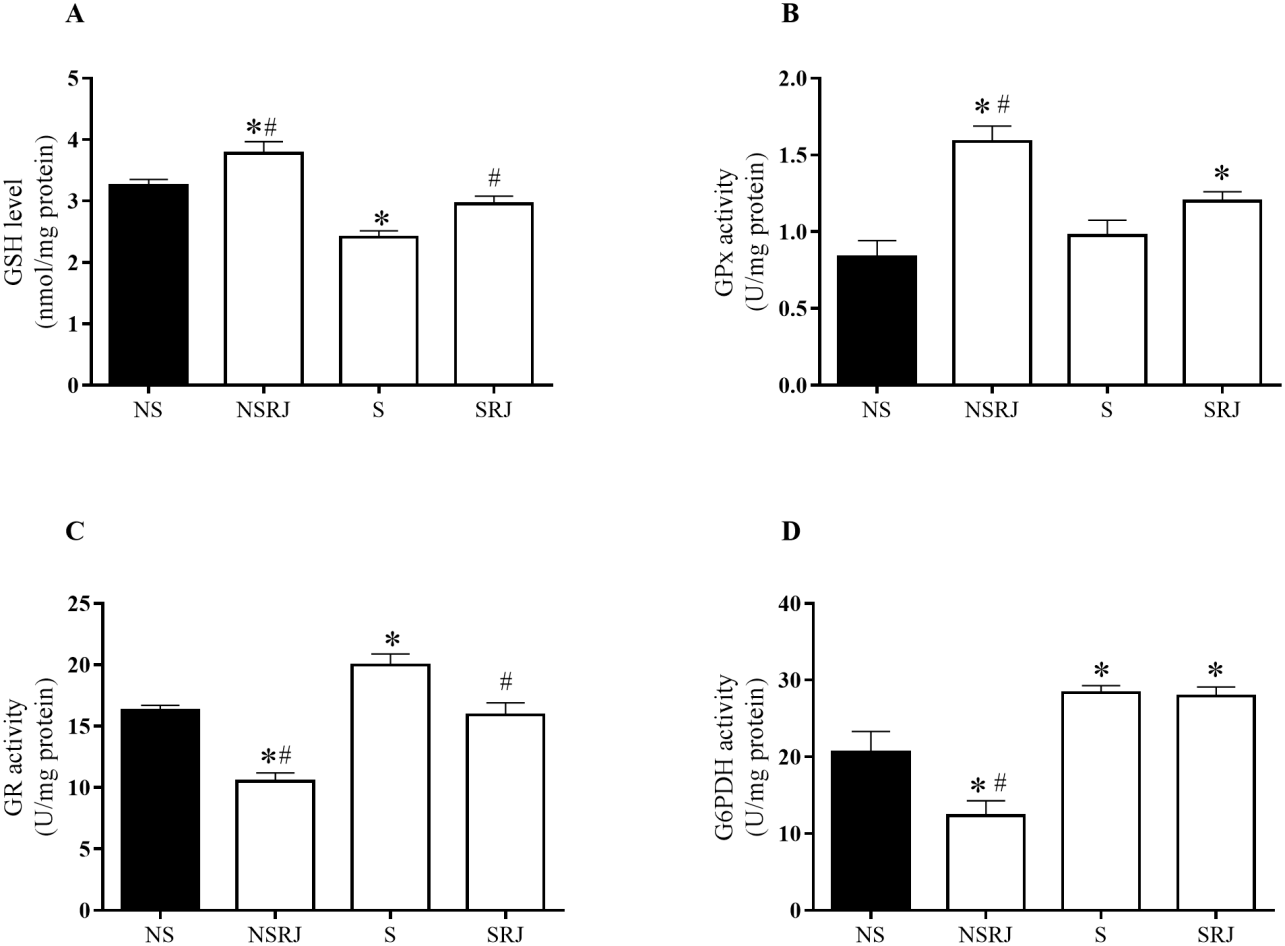
Glutathione antioxidant defense system in liver tissue of rats stressed by immobilization and cold. Glutathione level (GSH) (A). Glutathione peroxidase activity (GPx) (B). Glutathione reductase activity (GR) (C). Glucose-6-phosphate dehydrogenase activity (G6PDH) (D). No stress (NS), No Stress supplemented with Royal Jelly (NSRJ), Stress (S) and Stress supplemented with Royal Jelly (SRJ). Values are expressed as mean±SEM, * p < 0.05 vs. NS, # p < 0.05 vs. S (One-way ANOVA followed by Tukey teste). Outliers were detected by performing Grubb’s test using an online GraphPad outlier calculator (http://graphpad.com/quickcalcs/Grubbs1.cfm).

The glutathione defense system analysis in liver tissues is displayed in Fig 4. GSH content was lower in S compared to NS (F_3, 51_ = 17.65, p < 0.001), whereas GR and G6PDH levels were higher in S compared to NS (F_3, 21_ = 20.42, p < 0.05; F_3, 20_ = 17.24, p < 0.05, respectively). No significant difference was observed in GPx activity in S rats compared with NS rats. In addition, the glutathione defense system analysis of the RJ supplemented S rats showed an increase in GSH content (F_3, 51_ = 17.65, p < 0.05), a decrease in the GR level (F_3, 21_ = 20.42, p < 0.05), whereas no difference was observed in GPx and G6PDH activity compared to S rats.

## Discussion

Studies have been carried out to assess agents that could prevent the damage to liver tissues triggered by acute and chronic stress, which induce the formation of ROS followed by hepatic injury [26]^,^[27]. In this study, the adaptogenic potential of RJ was assessed and associated with the antioxidant activity and anti-stress capacity, to improve the organism’s adaptation to stress induction and in absence of stress. Our results showed a decrease of corticosterone and blood glucose levels, weight loss as well as an improvement in the antioxidative parameters in the liver of stressed RJ-supplemented rats.

Stress promotes the activation of the HPA axis, thus stimulating the release of corticosterone by the adrenal gland [3]. This increase in corticosterone mobilizes energy substrates in liver tissues to maintain homeostasis in a stress situation [28]. In the present study, restraint and cold stress not only augmented corticosterone levels, blood glucose levels and biomarkers of oxidative stress, but also diminished the glutathione antioxidant defense system in the liver tissues. Our results corroborate other studies that reported an increase of corticosterone levels under several stress models such as restraint and cold, immobilization and cold, besides other models of stress induction [2, 4–6, 20, 29]. Besides that, we showed a decreased of the corticosterone level associated with RJ supplementation, even in absence of stress. Teixeira et al. (2017) also demonstrated the effect of RJ in reducing corticosterone levels in stressed rats [20]. Corticosterone synthesis is cholesterol-dependent, which indicates the possibility that RJ is able to inhibit the synthesis of cholesterol. Major royal jelly protein 1 was identified as a hypocholesterolemic protein [30], indicating a possible mechanism through which RJ could inhibit corticosterone synthesis. Furthermore, the decreased corticosterone levels improved glucose uptake [31], which could control glycemia even after stress induction.

Fontella et al. (2005) demonstrated that the repeated exposure of adult rats to restraint stress causes a temporary suppression of food intake and reduction of body weight [32]. Herein, we observed a decrease of body weight in both groups of stressed rats and non-stressed rats supplemented with RJ, even without a diminution in food intake. These results corroborate a prior study [32] demonstrating that the diminution of body weight in restriction and cold stressed rats is attributable to an elevation of corticosterone levels. Furthermore, our data indicate that the RJ can also diminish body weight even in the absence of stress induction. Although we did not evaluate the mechanism by which supplementation with RJ reduces body weight, other studies have also showed that RJ supplementation can diminish body weight [33, 34]. New studies must be conducted to investigate the mechanism by which this effect occurs.

Stress alters the availability of metabolic substrates and can influence blood glucose levels, leading to an increase in oxidative stress [35]. Our results indicate that the glycemia of non-stressed rats supplemented with royal jelly did not differ from the non-stressed group. Although we did not measure the plasmatic insulin, a prior study demonstrated that RJ did not affect insulin levels [36]. Thus, the augmentation of corticosterone levels is probably responsible for increased the blood glucose level under this stress situation, due to its role in hepatic gluconeogenesis [37]. Furthermore, a strong positive correlation between glycemia and corticosterone levels was observed after the last stress induction session. These results indicate not only that glycemic control is corticosterone-dependent in the stress response, but also that after the last stress session (Sa and SRJa) the RJ supplementation diminished the increase of blood glucose levels compared to Sa rats. Thus, the capacity to inhibit the increase in blood glucose levels after stress induction indicates a potential anti-stress and adaptogenic effects of RJ.

Corticosterone acts on the liver, increasing glucose production especially through gluconeogenesis [37]. It can also increase oxidative stress and induce damage in the liver tissue [38]. Stressed rats presented increased plasma GGT compared with NS rats, indicating hepatic damage [39]. RJ supplementation reduced both GGT levels and AST compared with S rats, and also reduced AST and ALP compared with NS rats. Other studies using models of toxicity induced by lambda-cyhalothrin and azathioprine also found a decrease in these enzymes, supporting the protective effect of RJ towards liver tissue [40, 41]. Thus, data observed here suggests a hepatoprotective effect of RJ, not only by improving the liver enzymes, but also the antioxidative systems.

High levels of glucocorticoids and exposure to stress increase ROS [10, 11]. Herein, stressed rats had increased lipid peroxidation associated with the corticosterone level, corroborating other studies [38, 42]. However, RJ supplementation decreased the lipid peroxidation in the liver of stressed rats. RJ peptides can diminish the peroxidation of linoleic acid and eliminate hydroxyl radicals, inhibiting lipid peroxidation [43]. In addition, in the present study, stressed rats decreased total antioxidant capacity associated with the increase of corticosterone levels, whereas RJ supplementation restored FRAP to levels comparable to those of NS rats. RJ contains vitamins B and E, zinc, copper [44], phenolic compounds [45] and peptides with antioxidant actions [43]. These RJ compounds can act as antioxidant agents preventing the oxidative damage in the liver, suggesting that RJ supplementation may improve the antioxidant defense of stressed rats.

Furthermore, we analyzed the enzymatic and glutathione antioxidant defense system in the liver tissues of the stressed rats. SOD activity decreased in stressed rats compared with NS and increased in rats supplemented with RJ compared with S rats. Other studies have also shown a decrease in this enzyme activity in the liver of rats undergoing stress, thereby corroborating our results [46, 47]. Oishi and Mashida (2009) reported a decrease in hepatic SOD mRNA six hours after stress by immobilization and cold [48]. Furthermore, our findings of decreased GSH and increased GR and G6PDH activities in stressed rats, indicate a compensatory mechanism to maintain the redox cycle of GSH, as shown in other studies [11, 42].

RJ supplementation also increased the GSH level, corroborating Karadeniz et al. (2011), who showed that RJ-supplemented rats present increased GSH in the liver and kidney in an oxidative stress model induced by cisplatin [49]. Furthermore, RJ supplementation decreased GR activity compared to S rats, and increased GPx and G6PDH activities similar to NS rats. Studies employing models of toxicity for cisplatin [49] and paracetamol [50] also showed increased GPx activity in rat livers. The unchanged GPx activity between stressed rats shown in the present study was not found in previous studies. Our finding may be attributable to the stress model used in this study. To the best of our knowledge, this is the first study that has observed the effect of RJ supplementation on the activity of GR and G6PDH in the liver during a stress situation. The RJ antioxidant property may be derived from the short-chain peptides [43], phenolic compounds (flavonoids and cinnamic acid derivatives) [45], some antioxidant type vitamins (A and E) and fatty acids (trans-10-Hydroxy-2-decenoic) [19]. Therefore, these results support the antioxidant activity of RJ and indicate a prevention of hepatic oxidative damage caused by stress.

## Conclusion

RJ decreases corticosterone and improves glycemia control after stress induction. Moreover, RJ showed a hepatoprotective effect against oxidative damage, reducing lipoperoxidation and increasing the total antioxidant capacity in liver tissues of restraint and cold stressed rats. Taken together, these results highlight an adaptogenic role of RJ in situations of stress and oxidative damage.
